# Extension Services for Livestock Keepers in Low-Income Countries—A Low Priority?

**DOI:** 10.3390/ani12060726

**Published:** 2022-03-14

**Authors:** Salimata Pousga, Ulf Magnusson, Ismail Moumouni, Guiguigbaza-Kossigan Dayo, Assa Kante, Sofia Boqvist

**Affiliations:** 1 Institut du Développement Rural, Université Nazi Boni, Bobo-Dioulasso BP 1091, Burkina Faso; 2 Department of Clinical Science, Swedish University of Agricultural Science, 750 07 Uppsala, Sweden; ulf.magnusson@slu.se; 3 Département d’Economie et de Sociologie Rurales, Université de Parakou, Parakou BP 123, Benin; mmismailfr@yahoo.fr; 4 Centre International de Recherche, Développement sur l’elevage en zone Subhumide (CIRDES), Bobo-Dioulasso BP 454, Burkina Faso; charlesdayo@yahoo.fr; 5 Sassakawa Africa Fund for Extension Education (SAFE), Sassakawa Africa Association (SAA), Bamako BP E3541, Mali; kante.assa@gmail.com; 6 Department of Biomedical Sciences and Veterinary Public Health, Swedish University of Agricultural Science, 750 07 Uppsala, Sweden

**Keywords:** West Africa, food security, animal sourced food, small holder, pastoral, agro-pastoral

## Abstract

**Simple Summary:**

Animal-source foods are an important dietary complement to the calories in staple food, but in low-income countries, productivity on the smallholder farms that provide most of the domestic food supply is low. The general objective of this survey-based study was to contribute to effective integration of livestock issues in agricultural extension and advisory programs within the framework of sustainable food and nutrition security in Burkina Faso, Mali, and Benin. The findings show that despite the equal importance given by farmers to animal and plant production, livestock production appears to be disadvantaged in terms of access to extension services and new technology compared with plant production, even though many farmers are willing to pay for this service if available. Furthermore, livestock farming is facing constraints related to feeding, health, and reproduction, limiting development of the sector. Based on this study, we recommend that agricultural extension programs be planned in a holistic context, taking into account the major concerns of farmers, with technological packages in integrated crop-livestock systems.

**Abstract:**

Achievement of sustainable agricultural development and national food security in Africa is dependent on several factors, including productivity in the livestock production sub-sector. This study surveyed farmers’ perceptions on provision of extension services relating to livestock production in Burkina Faso, Mali, and Benin. A structured questionnaire comprising dichotomous, multiple-choice, and open-ended questions was used to survey a total of 1560 farmers in Burkina Faso, 345 in Mali, and 480 in Benin. Most farmers surveyed pursued integrated crop and livestock production, but more frequently in Burkina Faso (91%) than in Mali and Benin (66%). Around one-third (36%) of the respondents in Burkina Faso had access to livestock extension services, while the corresponding figure in Mali and Benin was 54% and 69%, respectively (*p* < 0.01). Moreover, 71% of respondents in Mali, 73% in Burkina Faso, and 84% in Benin reported significantly (*p* < 0.05) fewer extension activities for livestock compared with crop production. Thus, livestock production seems to be given low priority in agricultural extension interventions. We recommend that future diffusion of technological packages should be more holistic, considering the major concerns of the specific environment and the socio-cultural traditions of both livestock and crop producers.

## 1. Introduction

It is well-established that livestock play a critical role for livelihoods, and food and nutrition security in low-income countries [1,2]. Achieving several of the United Nations Sustainable Development Goals (SDG) will depend on their ability to establish sustainable livestock production to meet the needs of a growing population. Production of livestock in low-income countries is widely acknowledged as a pathway out of poverty, a major income-generating activity, and a means of income diversification [1,3]. Agricultural extension is important for building capacity among livestock farmers in low-income countries. It includes transfer of information and technology from the global knowledge base and local research to farmers, enabling them to identify their own goals and possibilities, helping them to improve their productivity and profit, and stimulating agricultural development. Targeted agricultural extension can be a way to increase sustainable food production and improve livelihoods in such settings [4]. Supporting local food production may be one important measure to curb the increase in undernutrition in people world-wide, which has accelerated in recent years [5]. As well as contributing to overall food security, animal-source foods provide essential micronutrients for good health and quality of life, and are thus an important complement to the calories in staple food [2,6,7]. High productivity in the livestock sector is an integral part of sustainable agricultural development and national food security in Africa [1,8]. Livestock not only provide nutritious food, but also support livelihoods by producing high-value food commodities for sale, increasing agricultural yield through enhancing soil fertility (organic manure), and providing draft power that enables tillage of larger areas [9].

In West Africa, farming systems generally combine crop and livestock farming [10]. However, livestock farming mainly involves low-skilled farmers rearing animals on a subsistence basis. Livestock extension services in West Africa to date are mainly based on providing information on animal management at a local level and at animal health clinics or camps [11]. Studies from sub-Saharan Africa (SSA) show that farmers perceive there are fewer extension services targeting livestock production compared with crop production [12,13]. It has also been shown that small scale livestock farmers perceive challenges related to the quality of extension services provided and that they are inclined to take advise from non-extension services [12,14]. There is thus a critical need for livestock health education and training for pastoralists as well as for extension officers [15]. Extension services play important roles to facilitate dissemination of science-based knowledge to livestock farmers. This might be challenged due to lack of efficient communication between research institutes, extension services and end-users which prevent science-based knowledge to be translated into practice [16]. Furthermore, extension services need to be context-specific to contribute to development of the livestock sector in, e.g., SSA, and there is a paucity in data on livestock farmers information needs, information sources and pathways, as well as about the farm typology to allow for targeted extension [12,15,17].

The aim of this study was to map farmers’ perceptions of extension services provision for livestock producers in three West African countries, Burkina Faso, Mali, and Benin, and to assess how this compares with extension services provision for crop producers.

## 2. Materials and Methods

### 2.1. Study Area and Livestock Systems

The study was carried out in Burkina Faso, Mali, and Benin in West Africa. The climate type in the region is Sahelian, with an arid to semi-arid climate in Mali, a semi-arid climate in Burkina Faso, and a tropical humid climate in Benin. The land area of Burkina Faso, Mali, and Benin is around 273,000, 1,000,000, and 113,000 km^2^, respectively [18]. All three countries, and most other West African countries, have an economy based on the agricultural sector, with primary production located mainly in rural areas [19]. There are two main livestock systems (agro-pastoral and pastoral) within the three countries. The agro-pastoral system is based on integration of livestock activities into agricultural activities, and livestock can be kept in pastoral areas in periods of few agronomic activities (plowing, sowing, harvesting, crushing cereals) [20]. The pastoral system predominates in areas with unsuitable conditions for crop production. The main characteristics of pastoral systems are mobility of herds and low inputs [20].

### 2.2. Selection of Farms

The present study aimed to cover all regions in each country (13 in Burkina Faso, 11 in Mali, 12 in Benin). However, in Mali selection of regions had to be adjusted for security reasons and, instead, three provinces within six regions in the central part of the country and in the south (Bamako, Kayes, Koulikoro, Sikasso, Ségou, Mopti), and one province in five high-risk security regions (Gao, Ménaka, Tombouctou, Taoudéni, and Kidal) in the north were selected. In each of these provinces, three communes were included based on travel safety. In all three countries, farms in the villages listed in the records of the relevant rural development ministry were considered for selection. The local agricultural extension agents, together with the person responsible for livestock vaccination in the village and project members in each country, were involved in the selection of farms.

In Burkina Faso, staff from the Ministry of Animal and Fishery Resources assisted in the selection of farms. In each of Burkina Faso’s 13 regions, two provinces were selected based on accessibility, and two villages per province were selected based on farmers’ involvement in livestock rearing. Thirty livestock-owning farms per village were then randomly chosen for the survey, in consultation with the person responsible for livestock vaccination in the village and the enumerator in the project. This resulted in a total number of 1560 farms being selected for the study. In Mali, the selection of farms was performed in collaboration with staff from the National Directorate on Livestock Development within the Ministry of Rural Development. In total, 69 communes were selected in the 23 provinces and five livestock-owning farms were randomly selected in each commune, giving a total of 345 farms. In Benin, farms were selected in collaboration with the Directorate of Animal Production. From each region, two communes were selected based on accessibility (24 communes in total). In each commune, two villages were selected based on previous experience of agricultural extension services. From each of these 48 villages, 10 farms were selected randomly for the study based on livestock ownership, which gave a total of 480 farms.

Prior to the field survey, in each country visits were made by the local project team members to discuss the study with the village development committee in each selected village. Furthermore, selected farms were only included in the study if a household member with responsibility for the livestock in the household was at home and willing to participate. All farmers were informed that participation in the survey was voluntary and that all data would be handled anonymously. Local project team members consisted of staff from Nazi Boni University and the Ministry of Animal Resources in Burkina Faso, staff from the University of Segou, the Sassakawa Association, the Rural Polytechnic Institute of Katibougou and the Ministry of Rural Development in Mali, and staff from the University of Parakou and the Ministry of Rural Development in Benin.

### 2.3. Data Collection

A structured questionnaire containing a combination of dichotomous, multiple-choice, and open-ended questions was developed. The questions covered demographics, socio-economic characteristics, livestock management, and the respondent’s experiences and perceptions about livestock extension services. The questionnaire was pre-tested in farms in peri-urban areas of Bobo-Dioulasso (Burkina Faso), Bamako (Mali), and Parakou (Benin), and was refined based on inputs from these pre-testing sessions. The final questionnaire (available from the first author), took about 30 min to complete. The interviews were held in the native languages Dioula and Moore in Burkina Faso, Bambara in Mali, and Bariba, Peulh, Mahi, Nagot, and Fon in Benin, and were carried out with the assistance of the same interpreter for each language throughout the study. The interviews were carried out by trained members of the project team.

### 2.4. Selection of Extension Service Institutions

Extension services within the three study countries were contacted to get information about whether they were involved in livestock extension and if so, in what way. In Burkina Faso, the extension institutions contacted were selected in collaboration with the Directorate of Actor Capacity Building at the Ministry of Animal and Fishery Resources. In Mali, the selection process was facilitated by the General Directorate of Sasakawa Africa Fund for Extension Education (SAFE) in Francophone countries, and in Benin by the Directorate of Agricultural Advisory and Operational Training. A total of 15 extension institutions were contacted in Burkina Faso, 19 in Mali, and 7 in Benin.

### 2.5. Data Analysis

Data from each country were analyzed using Epi Info (Epi InfoTM 7.2.4.0; CDC, Atlanta, GA, USA) and Minitab (Minitab 16 Statistical Software, State College, State College, PA, USA: Minitab, Inc.) for calculation of frequencies, mean values of numerical data, and statistical parameters. Cross-tabulation and Chi-Square test were used to check for statistically significant associations between categorical variables (countries versus parameters). A value of *p* < 0.05 was considered significant.

## 3. Results

### 3.1. Demographics, Farm Characteristics, and Consumption of Livestock-Source Food

In total, 1560 farms were included in Burkina Faso, 345 in Mali, and 480 in Benin, and 99% of the farmers approached in Burkina Faso, 90% of those in Mali, and 91% of those in Benin participated in the study. More than 75% of the responding farmers were male and many had no formal education (63% of farmers in Burkina Faso, 38% in Mali, and 54% in Benin) (Table 1). There was a significant association between country and level of education (*p* < 0.01). The mean age of participating farmers was 46 years in Burkina Faso and 43 years in Benin (Table 1). Data on farmers’ age were not obtained in Mali, as most of the respondents did not want to provide information on their age. The data also showed that most of the participating livestock farmers had an agro-pastoral system (91% in Burkina Faso, 66% in Mali and Benin), with a significant association between country and production system (*p* < 0.01) (Table 1).

In Mali and Benin, the main reason cited for keeping livestock was for income generation (94% and 90%, respectively), while in Burkina Faso, the main reason was for crop production (soil fertilization and draft power) (84%) (Table 1). There was a significant association between country and frequency of animal products in the diet (*p* < 0.01): in Mali, 77% of participating farmers reported consuming animal products every day, compared with 17% in Benin and only 1.6% in Burkina Faso (Table 1).

### 3.2. Farmers’ Perceptions of Agricultural Extension Services Provision in Their Country

The farmers who responded to the questions relating to provision of agricultural extension services represented 98% of participating farmers in Burkina Faso, 88% in Mali, and 91% in Benin. There was a significant association between country and provision of farmers with access to livestock extension services (*p* < 0.01). Of the respondents in Burkina Faso, 36% reported that they had access to livestock extension services, while the corresponding figure in Mali and Benin was 54% and 69%, respectively (Table 2).

Of those that had received training through livestock extension services, 81% of participating farmers in Burkina Faso reported having accessed extension services related to animal health. The corresponding figure in Mali was 49% and that in Benin was 60% (Table 2). The most common livestock-related extension service in Mali was related to feeding. Most farmers in all three countries received livestock extension services from the government (ranging from 58% in Burkina Faso to 80% in Mali), followed by externally funded project-based extension services. There was a significant association between country and extension service provider (*p* < 0.05) (Table 2).

### 3.3. Comparison of Extension Service Provision on Crop and Livestock Production

Farmers’ general perceptions on extension service provision were that there were fewer extension activities or programs for livestock production compared with crop production. This opinion was expressed by 71% of participating farmers in Mali, 73% in Burkina Faso, and 85% in Benin (*p* < 0.05) (Table 3). Between 69% (Mali) and 89% (Burkina Faso) of participating farmers also reported having less access to livestock-related technologies than crop farmers (*p* < 0.01). Furthermore, between 70% (Mali) and 79% (Benin) of participating farmers from the three countries perceived there were fewer farmers’ organizations targeting livestock farmers than were available for crop farmers. Among the participating farmers, 44% in Burkina Faso, 55% in Mali, and 41% in Benin also believed that market access was less developed for livestock and livestock products compared with crop and crop products. Participating farmers’ perceptions on support for livestock and crop production in terms of extension activities or services are summarized in detail in Table 3.

### 3.4. Farmers’ Demand for Livestock Extension Services and Actual Provision of Such Services

The vast majority (99%) of respondents in Burkina Faso and in Mali, and 90% in Benin, expressed a desire for more livestock extension services. Table 4 lists the animal production fields in which participating farmers reported willingness to pay for extension services and training. Notably, the majority of participating farmers in the three countries were willing to pay for livestock extension services to improve animal health.

In Burkina Faso and Mali, 40% and 42%, respectively, of the extension service providers contacted in the present study reported providing services relating to the livestock sector. In Benin, all seven agricultural extension services contacted reported providing services relating to livestock. The characteristics of these livestock extension services providers are summarized in Table 5.

## 4. Discussion

This study revealed a need for increased provision of livestock-related extension services in Burkina Faso, Benin, and Mali. The majority of participating farmers perceived that there were fewer extension services targeting the livestock sector compared with the crop sector. This is in accordance with previous findings by Fetch et al. [13] for Malawi, where the most common agricultural advice provided to farmers was on introduction of new methods of cultivation and new crops, crop fertilizers, and obtaining new seeds. Similar findings have been reported for Ethiopia [12].

The present study showed that government sources dominated the existing extension service provision within the livestock sector, followed by externally funded projects. Government extension agents were also the main source of agricultural extension services in a recently published study in Malawi [13]. It could be claimed that government-supported extension services are more important in the long term, as project-funded extension services may cease when the project ends. In the study in Malawi [13], the media were the second largest source of extension services. The role of the media was not specifically investigated in the present study, but it was mentioned as being one of several extension services provided.

Data obtained in the survey showed that more than 75% of responding livestock-farmers in the three countries were male. This is typical for Africa, as traditionally women are not in charge of farming but are the main actors in small ruminant, poultry, and micro-livestock production, as well as in dairying [21,22]. Even when females participate in training, they may not be given equal recognition for their responsibilities and skills, and female farmers may have less access to extension services and may also face cultural barriers [12,23]. This should be considered when developing livestock extension services [24]. The survey results also showed that the majority of livestock farmers in all three countries integrated crop and livestock production (agro-pastoral system), as reported previously for other West African countries. For example, Iyiola et al. [25] found that 80% of farmers in Nigeria practiced combined crop and livestock production. In the three countries included in the present study, between 15 and 40% of national GDP is generated within the livestock sector [26,27,28], which is in alignment with the economic importance of livestock in other low- and middle-income countries [1,29]. The importance of livestock production for national GDP is likely not reflected in the amount of extension services provided for livestock farmers surveyed in the present study.

The frequency of consumption of animal products by responding farmers in the survey countries was low, particularly in Burkina Faso. The agro-pastoral system, where the animal plays a crucial role as draft power and contributes to intensification of cropping systems (manure as fertilizer), as well as a source of cash income (sale as market product) rather than self-consumption of products [20], is likely one reason for this. There might also be the case that cultural and religious beliefs place restrictions on the animal-source foods that may be eaten, and by whom [6]. Our results confirm findings in a previous study that recommended consumption levels (13.7 kg meat and 40 L milk per person and year) are far from being met at national level in sub-Saharan Africa in general and in Burkina Faso in particular [30]. The pastoral system is characterized by relatively low involvement in market systems due to, e.g., long distance from markets, low inputs, and high self-consumption of animal products [20]. Long distances may also contribute to poor access to extension services within the livestock sector [15].

Responding farmers appeared to attribute importance to both animal and crop production in all three countries surveyed, as shown by the high percentage using the agro-pastoral system. However, despite this dual importance, the data also suggested that livestock production is disadvantaged in terms of access to technology. In Burkina Faso in particular, farmers were more likely to use an agro-pastoral system (91%) compared with Mali and Benin (66%). This implies that agricultural extension service providers are not giving the same importance to both animal and crop production in all three countries.

Livestock extension services were generally reported to be less common in Burkina Faso compared with Mali and Benin. The importance of livestock extension services has been shown by Adisa et al. [8]. In their study in Nigeria, inadequate extension services, low technological inputs, and poor communication (and utilization) of livestock research findings were found to be the major causes of prevailing low productivity in the livestock sector. A strong role of agricultural extension and training services in influencing uptake of best practices by livestock farmers has been demonstrated in Europe [31]. Thus, more agricultural extension and training work is needed in West Africa to ensure adoption of innovations by local farmers.

Extension workers in Africa face many challenges: for example, resource limitations including lack of transportation and diagnostic supplies, which limit access to livestock health services [15]. There may also be a lack of coordination between the different extension service providers. In addition, there are few or limited financial resources available for providing training for livestock producers, although the majority of these contribute financially, as shown in this study. Different sources of agricultural advice were mentioned in this study, but none included use of information and communications technology (ICT), such mobile phones and the internet. There is untapped potential in using ICT as a way to transform existing extension systems in low-income countries [32,33]. Such a transformation could contribute to increased access to extension services in the three countries surveyed here and make it more robust. However, sources of agricultural extension services are context-specific and consideration must be taken of farmers’ preferences, illiteracy, and access to technology [12]. Furthermore, development policies in rural areas seem to prioritize crop production, even though livestock production is equally important for food and nutrition security in the population and may serve as a way out of poverty [34]. It is possible that agricultural policies at government level do not translate into programs and interventions contributing to improvements at farm level [13].

## 5. Conclusions

This survey of smallholder livestock farmers in three West African countries (Burkina Faso, Mali, and Benin) revealed that most of the surveyed farmers combined crop and livestock production. Livestock production was perceived to be disadvantaged in terms of access to extension services and new technology compared with plant production. This was despite that many farmers were willing to pay for this service if available. Livestock farming was facing several constraints, for example, related to animal feeding and health, likely limiting development of the sector. Thus, to improve rural livelihoods and secure access to nutritious diets in the surveyed countries, more emphasis should be placed on developing professional, accessible, and affordable advisory and extension services for livestock farmers. However, sources of agricultural extension services are context-specific and consideration must be taken of farmers’ preference, illiteracy, and access to technology relevant for the particular farm type. This study provided valuable insights on the needs of livestock farmers in Burkina Faso, Mali, and Benin for better livestock extension services. These insights can be used to plan more targeted extension interventions to support livestock production in integrated crop-livestock systems.

## Figures and Tables

**Table 1:** General characteristics of participating farmers in Burkina Faso, Mali, and Benin, and their livestock production system and frequency of meat consumption.

	Burkina Faso (%)	Mali (%)	Benin (%)	*p*-Value
Gender:				<0.01
Male	76	94	89	
Female	24	6	11	
Mean age in years (min–max)	56 (18–85)	NA^1^	43 (19–70)	
Level of education:				<0.01
No education	63	38	54	
Primary school	14	25	25	
Secondary school	5	11	15	
University	0.14	1.5	3.7	
Other	18	24	3	
Livestock production system:				<0.01
Pastoral system	9	34	34	
Agro-pastoral	91	66	66	
Reasons for rearing animals:				<0.01
Financial insurance	0.13	47	16	
Tradition	0.82	42	25	
Income generation	8.4	94	90	
Household consumption	5.2	35	32	
Draft power/fertilizer	84	66	NA	
Other	0.6	4	27	
Frequency of meat consumption:				<0.01
Every day	4	65	73	
Once per week	22	15	6.6	
Three times per week	24	8.7	5	
Once per month	32	5	9.4	
Special occasions	18	6.4	6	

**Table 2:** Proportion of respondents in Burkina Faso, Mali, and Benin that reported receiving livestock extension services, and the service provider/s.

	Burkina Faso (%)	Mali (%)	Benin (%)	*p*-Value
Have received livestock extension services on:	36	54	69	<0.01
Equipment/materials ^1^	9	15	10	
Training on reproduction ^1^	1.6	18	4	
Training on feeding ^1^	24	75	26	
Health management ^1^	81	49	60	
Farm management ^1^	3	31	24	
Vaccination campaign ^1^	1.5	70	14	
Have not received livestock extension services	64	46	31	
Livestock extension service provided				<0.05
Government	58	80	66	
Externally funded project	42	39	14	
Local NGO	0.2	18	7	
Foreign NGO	-	11	4	
Private veterinarian	0.2	17	1.6	
Other	-	13	36	
Reasons for not getting livestock extension services				
No interest	0.27	34	8.7	
Had asked, but did not get it	3	29	17	
Did not know where to get it	96	37	21	

^1^ Of those who received livestock extension services.

**Table 3:** Perceptions of respondents in Burkina Faso, Mali, and Benin on provision of extension services relating to livestock production, compared with the provision relating to crop production.

	Burkina Faso (%)	Mali (%)	Benin (%)	*p*-Value
State of extension activities on livestock:				<0.01
There are fewer extension activities on livestock	73	71	85	
Extension activities are the same	24	6.2	13	
There are more extension activities on livestock	2.8	23	2.7	
Access to technologies in livestock farming:				<0.01
Livestock farmers have less access to technologies	89	69	77	
Access to technologies is the same	6.7	30	17	
Livestock farmers have more access to technologies	3.9	0.6	5.9	
Access to information in livestock farming:				<0.01
Livestock farmers have less access to information	66	61	77	
Access to information is the same	29	20	18	
Livestock farmers have more access to information	4.9	19	5.9	
Farmers’ organizations:				<0.01
There are fewer organizations for livestock farmers	76	70	79	
There are similar numbers of organizations for livestock and crop farmers	20	6.7	15	
There are more organizations for livestock farmer	4.3	23	6.2	

**Table 4:** Animal production fields in which farmers in Burkina Faso, Mali, and Benin were willing to pay for extension and training services.

Parameter	Burkina Faso (%)	Mali (%)	Benin (%)	*p*-Value
Animal production field:				<0.01
Animal health	92	69	56	
Animal reproduction	1.6	46	51	
Animal nutrition	5.6	61	50	
Hygiene and housing	0.1	11	30	
Equipment	0.4	20	27	

**Table 5:** Characteristics of the livestock extension services provided by extension and advisory institutions in Mali, Burkina Faso, and Benin contacted in this study.

Livestock Extension Services Provided	Objectives of the Livestock Extension Service
Livestock farm visits and technical meetings	Encourage, advise, and support farmers Evaluate technical support and innovations Assess livestock production performance
Demonstration units/farmer school/participatory, technology development	Promote adoption and implementation of technologies in animal husbandry Set up extension tools at village level Promote technologies and learn how they are used
Outreach/communication ^1^	Promote livestock products and technologies Reward and encourage actors in the livestock sector Raise awareness among stakeholders in the animal and fishery sectors
Innovation platforms in West Africa	Create and support network for researchers, extension service providers, and policy makers

^1^ For example: radio and television broadcasting, exhibitions and fares, performances.

## Data Availability

Data can be obtained by contacting the first author.
